# Endophytic Fungi Isolated from *Ageratina adenophora* Exhibits Potential Antimicrobial Activity against Multidrug-Resistant *Staphylococcus aureus*

**DOI:** 10.3390/plants12030650

**Published:** 2023-02-01

**Authors:** Juan Wen, Samuel Kumi Okyere, Jianchen Wang, Ruya Huang, Ya Wang, Lin Liu, Xiang Nong, Yanchun Hu

**Affiliations:** 1 Key Laboratory of Animal Disease and Human Health of Sichuan Province, Sichuan Agricultural University, Chengdu 611130, China; 2 Department of Pharmaceutical Sciences, School of Medicine, Wayne State University, Detroit, MI 48201, USA; 3 College of Grassland Science and Technology, Sichuan Agricultural University, Chengdu 611130, China; 4 College of Life Science, Leshan Normal University, Leshan 614000, China; 5 New Ruipeng Pet Healthcare Group Co., Ltd., Shenzhen 518000, China

**Keywords:** *Ageratina adenophora*, antibacterial activity, endophytic fungi, LC-MS analysis, secondary metabolites

## Abstract

Multidrug-resistant bacteria such as *Staphylococcus aureus* (MRSA) cause infections that are difficult to treat globally, even with current available antibiotics. Therefore, there is an urgent need to search for novel antibiotics to tackle this problem. Endophytes are a potential source of novel bioactive compounds; however, the harnessing of novel pharmacological compounds from endophytes is infinite. Therefore, this study was designed to identify endophytic fungi (from *Ageratina adenophora*) with antibacterial activity against multidrug-resistant bacteria. Using fungal morphology and ITS-rDNA, endophytic fungi with antibacterial activities were isolated from *A. adenophora*. The results of the ITS rDNA sequence analysis showed that a total of 124 morphotype strains were identified. In addition, Species richness (*S*, 52), Margalef index (*D*^/^, 7.3337), Shannon–Wiener index (*H*^/^,3.6745), and Simpson’s diversity index (*D*, 0.9304) showed that *A. adenophora* have abundant endophytic fungi resources. Furthermore, the results of the agar well diffusion showed that the *Penicillium sclerotigenum*, *Diaporthe kochmanii*, and *Pestalotiopsis trachycarpicola* endophytic fungi’s ethyl acetate extracts showed moderate antibacterial and bactericidal activities, against methicillin-resistant *Staphylococcus aureus* (MRSA) SMU3194, with a MIC of 0.5–1 mg/mL and a MBC of 1–2 mg/mL. In summary, *A. adenophora* contains endophytic fungi resources that can be pharmacologically utilized, especially as antibacterial drugs.

## 1. Introduction

Fungi-derived natural products play an important role in the discovery of new drugs. Endophytic fungi live in the intercellular/intracellular regions of plant tissues without harming their host plant. Endophytic fungi are found in almost all plants in natural ecosystems and have complex ecological mutual relationships with their host plants [1]. In this mutual relationship, fungi synthesize bioactive secondary metabolites that promote the growth and development of the host plant, whereas the host plants serve as a habitat for these microorganisms. In recent years, molecular phylogenetics, metabolomics, and medicinal chemical analysis have made it possible to culture these endophytic fungi and isolate their bioactive compounds for biomedical purposes in vitro [2,3]. Furthermore, pharmaceutical natural products such as penicillin (which was the first antibiotic used in clinical practice and was isolated from *Penicillium notatum* cultures), podophyllotoxin, camptothecin, and analogs (which are antineoplastic agents synthesized by the endophytic fungi *Trametes hirsuta* and *Alternaria alternata*) that were isolated from various endophytic fungi have been reported recently [4,5,6,7]. Therefore, endophytes (endophytic fungi) are a new source of bioactive substances for the development of novel drugs [8].

Mount Luoji (27°24′07″–27°39′24″ N, 102°15′27″–102°24′30″ E) is located in the southwestern part of Sichuan Province, China. Mount Luoji has an astonishingly high biodiversity with a typical subtropical alpine monsoon climate [9]. Several medicinal plants, such as *Panax notoginseng* (Burkill) F. H. Chen ex C. H., *Fritillaria cirrhosa* D. Don, *Gastrodia elata*, and *Codonopsis pilosula* (Franch.) Nannf, are dominant in this area. *Ageratina adenophora* (Spreng.) R.M. King & H. Rob. is one of the typical plant resources found at Mount Luoji. *A. adenophora* is a perennial herb widely distributed in China, India, and Australia. This particular plant is used in ethnomedicine for the treatment of the common cold with wind-heat syndrome, insomnia, jaundice, irregular menstruation, and diabetes [10,11,12]. *A. adenophora* contains some pharmacological ingredients such as terpenes, flavonoids, steroids, phenylpropanoid, and their various derivatives. Among these compounds, 9-oxo-10,11-dehydro-agerophorone (Euptox A) was abundant in the leaf oil (about 23.41%) [13]. Euptox A has shown antibacterial, antineoplastic, and acaricidal activities in different pharmacological models, demonstrating its medicinal potential [14,15]. *Escherichia coli*, *Staphylococcus aureus*, and *Enterococcus faecalis* are sensitive to extracts of *A. adenophora* [16,17]. Modern medical studies have shown that volatile oils from *A. adenophora* destroy *S. aureus* by damaging the cell membrane and inhibiting bacterial protein expression and nucleic acid synthesis [18]. Numerous studies have reported that the production of these bioactive compounds is correlated with the endophytes contained in the plant tissues [19]. In addition, endophytic fungi have been reported as an alternative source of bioactive compounds for future pharmacological drug development as they overcome certain challenges, such as slow-growing or rare and endangered plants, that are associated with the use of plant resources [20].

The widespread and frequent irrational use of veterinary and clinical antibiotics has resulted in a drastic increase in the number of multidrug-resistant pathogens. Methicillin-resistant *Staphylococcus aureus* (MRSA) is one of the important pathogens of hospitals and community-acquired infections worldwide, and is resistant to almost all β-lactam antibiotics used in animal and human treatments. MRSA colonization causes purulent skin infections, gastroenteritis, pneumonia, and urinary tract infections [21]. Furthermore, antibiotics developed in the last few years are ineffective against continuously evolving drug resistance systems in bacteria because of multidrug-resistance mechanisms, such as the synthesis of hydrolases and modifying enzymes, modification of active binding sites, decreased affinity of antibacterial drugs, decreased cell membrane permeability against drugs, and overexpression of efflux pump genes, which transport antibiotics out of the cell [22]. From a perspective of “One Health” strategy, developing new and effective antibiotic drugs is a global concern. The latest research advances in mycology have shown that about 51% of the secondary metabolites of fungi have unknown chemical structures, highlighting the biotechnological potential of these microbial communities as unique niches in the discovery of antibacterial drugs [23,24]. Numerous studies have also revealed the diversity of endophytic fungi in *A. adenophora*, but there is still a need for further exploitation of novel biomedical drug resources [25]. In this study, we isolated endophytic fungi strains from *A. adenophora* at Mount Luoji, China, analyzed their diversity, and evaluated their antibacterial activity. In addition, liquid chromatography–tandem mass spectrometry was used to analyze the bioactive compounds in the endophytic fungi extracts. To the best of our knowledge, this is the first study that systematically reported on the diversity of endophytic fungi in *A. adenophora*, their antibacterial activity, and the chemical compounds in their extracts. The findings from this study will add to the knowledge of the antibacterial potential of endophytic fungi and serve as the basis for the development of antibacterial drugs.

## 2. Results

### 2.1. Isolation, Identification, and Diversity of Culturable Endophytic Fungi in A. adenophora

**Figure 1:**
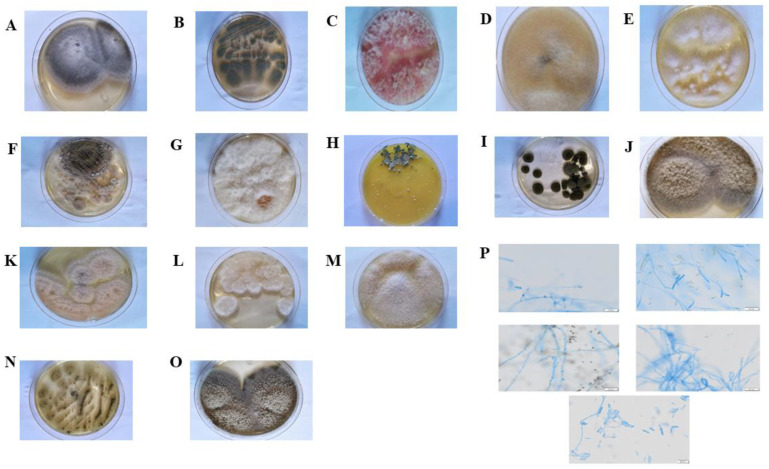
The morphology of various endophytic fungal colonies after incubation at 25 °C for 7 days: (**A**) *Cladosporium* sp. colony morphology showed brown or black colonies with dark-pigmented conidia; (**B**) *Phoma* sp. colony morphology showed brown chlamydospores that are arranged singly or in chains; (**C**) *Pestalotiopsis oryzae* colony morphology showed brown or dark brownish colonies with slimy conidial masses protruding from the surface; (**D**) *Mucor fragilis* colony morphology showed fluffy, white to dark-grey colonies with the development of sporangia; (**E**) *Botrytis cinerea* colony morphology showed whitish to greyish sclerotia colonies with short mycelium without sporulation; (**F**) *Phomopsis* sp. colony morphology showed white to brown hyphae and black fruiting bodies; (**G**) *Colletotrichum gloeosporioides* colony morphology showed white, fluffy, dense, light grey colonies with masses of conidia; (**H**) *Trametes versicolor* colony morphology shows whitish to light brown colonies with leathery texture; (**I**) *Fusarium graminearum* colony morphology showed pale or bright-colonies with a cottony aerial mycelium; (**J**) *Xylariaceae* sp. colony morphology showed yellow to brown colonies with scanty aerial mycelium; (**K**) *Diaporthe phaseolorum* colony morphology showed a white, lanose colony; (**L**) *Didymella* sp. colony morphology showed fluffy, whitish, grey colonies with aerial mycelia; (**M**) *Fusarium oxysporum* colony morphology showed whitish colonies with short conidia; (**N**) *Aspergillus flavus* colony morphology showed powdery masses of yellowish-green spores with cottony texture on the upper surface; (**O**) *Alternaria alternata* colony morphology showed green to olive-green colonies with small conidia; (**P**) Optical microscopy of mycelia of some endophytic fungi from *Ageratina adenophora* after incubation at 25 °C for 7 days.

In this study, a total of 212 fungal colonies were successfully isolated from 360 tissue segments of *A. adenophora* inoculated on a potato dextrose agar (PDA) medium. The 212 isolates were initially assigned to 124 representative morphotypes according to their colony morphology characteristics on PDA, and then later ITS rDNA sequences were subsequently generated for each morphotype (Figure 1 and Figure S2). *A. adenophora* endophytic fungi isolates were subjected to DNA extraction, PCR amplification, and sequencing. All the obtained sequences were subjected to Blast alignment and submitted to the NCBI GenBank database (Supplementary Table S1). Based on the sequence similarity threshold (SSA, 97–100%), 123 isolates were identified at the genus level, and the remaining isolates were unidentified. Based on Blast analysis results, 124 endophytic fungi strains were classified into 52 taxons (Table 1). Isolates DCL31 and DCL33 were classified as Basidiomycota, and isolates DCR10 and DCR14 were classified as Mucoromycota. In addition, the results of the diversity analysis showed that the values of Species Richness (*S*), Margalef index (*D^/^*), Shannon-Wiener index (*H^/^*), and Simpson’s diversity index (*D*) were 52, 7.3337, 3.6745, and 0.9304, respectively. These isolates were further classified into 3 phyla (Ascomycota, Basidiomycota, and Mucoromycota), 7 classes (Dothideomycetes, Eurotiomycetes, Sordariomycetes, Leotiomycetes, Pezizomycotina, Agaricomycetes, and Mucoromycetes), 12 orders, 19 families, and 25 genera of fungi (Table 2).

### 2.2. Antibacterial Activity

The antibacterial activity of endophytic fungi against multidrug-resistant gram-negative and gram-positive bacteria was determined in the crude extract of the endophytic fungi (Table 3 and Table 4).

As shown in Table 3, 25 endophytic fungi strains showed inhibitory activity against at least 1 type of antibiotic-sensitive bacteria, and 72% (18/25) of endophytic fungi showed broad-spectrum inhibition of gram-positive and gram-negative bacteria. Among them, five endophytic fungi strains: *Penicillium sclerotigenum* (DCL06), *Diaporthe kochmanii* (DCL09), *Pestalotiopsis trachycarpicola* (DCL44), *Fusarium solani* (DCR12), and *Penicillium ochrochloron* (DCR25) showed some antibacterial activity against all the multidrug-resistant gram-negative and gram-positive bacteria.

Furthermore, we determined the antibacterial activity of the five endophytic fungi extracts on veterinary multidrug-resistant gram-negative and gram-positive bacterial isolates. We found that among the five endophytic fungi, *P. sclerotigenum* (*Penicillium sclerotigenum*), *D. kochmanii* (*Diaporthe kochmanii*), and *P. trachycarpicola* (*Pestalotiopsis trachycarpicola*) extracts showed varying antibacterial activities against all multidrug-resistant bacteria strains, with MIC range of 0.5–2 mg/mL (Table 4). Concurrently, the extracts of these three fungi showed bactericidal activity against MRSA at a minimum bactericidal concentration range of 1–2 mg/mL. In comparison, none of the fungal extracts showed bactericidal activity against *S. agalactiae*.

**Figure 2:**
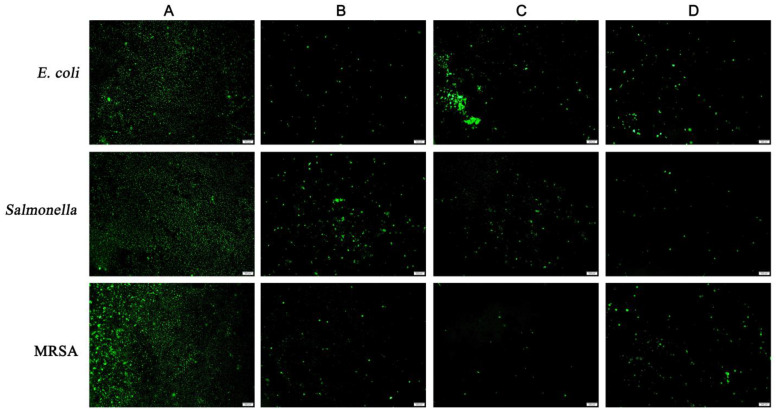
Effects of ethyl acetate extracts from the endophytic fungi on the cell membrane integrity of *E. coli, Salmonella,* and MRSA by fluorescence microscope. (**A**) Untreated bacterial cells; (**B**) bacterial cells treated with *P. sclerotigenum* extracts at 2MIC; (**C**) bacterial cells treated with *D. kochmanii* extracts at 2MIC; (**D**) bacterial cells treated with *P. trachycarpicola* extracts at 2MIC. The scale bar was 200 µm.

Acridine orange can freely penetrate cell membranes and specifically bind to nucleic acid in cells. Live cells in biofilms emit green fluorescence when stimulated with an argon laser. In addition to strong green fluorescence, cell membrane integrity was maintained in untreated cells (Figure 2A). In comparison with the untreated cells, the green fluorescence was significantly reduced after treatment with 2MIC ethyl acetate extract, indicating that ethyl acetate extract-induced cell membrane damage in veterinary and clinical multidrug-resistant pathogen isolates (*E. coli*, *Salmonella*, and MRSA) (Figure 2B–D). Therefore, we speculated that endophytic fungi ethyl acetate extract can disrupt cell membrane integrity to effectively inhibit bacterial proliferation and differentiation.

### 2.3. Liquid Chromatography—Tandem Mass Spectrometry

The ethyl acetate extracts of *P. sclerotigenum*, *D. kochmanii*, and *P. trachycarpicola* showed effective antibacterial activity. Therefore, Liquid chromatography–tandem mass spectrometry was used to analyze the chemical composition of the ethyl acetate extracts. Table 5 listed the names, retention time, molecular weight, molecular formula, mass-to-charge ratio, and content of compounds identified in extracts. Supplementary Figure S3 shows the chromatograms. Phenolic acids (caffeic acid, 2,3-dihydroxybenzoic acid), flavonoids (isorhamnetin, genistein, and taxifolin), fatty acids (malic acid, suberic acid), organic acids (citric acid, succinic acid, and phenyllactic acid), and monosaccharides (D-(−)fructose, sucrose, and xylitol) are among the compounds that were identified. As shown in Table 5, out of the 33 compounds identified from *P. sclerotigenum*; isorhamnetin was the primary compound, with a concentration of 6.21 µmol/g. In total, 23 compounds were identified in *D. kochmanii,* with phenyllactic acid (9.59 µmol/g) being the primary compound. Moreover, a total of 30 compounds were identified in *P. trachycarpicola*, with citric acid (21.14 µmol/g) and genistein (4.70 µmol/g) being the primary compounds. The levels of organic acids and flavonoids were the highest in the ethyl acetate extracts of the three endophytic fungi species, which could be attributed to the antibacterial activity of their crude secondary metabolites.

## 3. Discussion

Endophytic fungi are part of the microbial taxon with high taxonomic diversity [26]. These fungi can synthesize medicinal substances; hence, it could be an excellent source of novel antibacterial compounds for treating both human and animal pathogen infections [27]. However, studies on these microorganism resources are infinite and require the attention of microbiologists and medicinal chemists. Mount Luoji is rich in medicinal plant resources; however, few researchers have attempted to evaluate the diversity of these potential plant-associated endophytic fungi from these medicinal plant resources. Therefore, in this study, 124 morphotype strains belonging to 19 families and 25 genera were isolated from different *A. adenophora* tissues. Zhou et al. [28], similarly isolated *Diaporthe*, *Stagonosporopsis*, *Colletotrichum*, and *Alternaria* from *A. adenophora*. In this present study, we observed that some fungi species, such as *Nigrospora sphaerica* and *Mucor fragilis,* were only isolated from the roots, indicating that the endophytic fungi taxon distribution in the *A. adenophora* were organ- and tissue-specific. This specificity may be related to its anatomical structure and physiological conditions, which are generally consistent with many previous studies [29,30]. *Alternaria alternata* and *Cladosporium* sp. were found in various *A. adenophora* tissues, and their pleiotropic colonization may be related to the secondary metabolites secreted by them. These two fungi species are known to produce metabolites that protect host plants from herbivores and pathogens, as well as improve adaptation to abiotic stress [31]. In addition, we identified two new endophytic fungi, *Trametes versicolor* and *Ampelomyces* sp., which had not previously been reported in *A. adenophora* [32].

In the last three decades, multidrug-resistant bacterial infections have led to the search for novel compounds with broad-spectrum bioactivity, and endophytic fungi have been proven to synthesize effective antibacterial substances. The endophytic metabolites can be utilized for the development of antimicrobial agents [33]. In the present study, we found that the fermentation broth filtrates from 25 endophytic fungi strains, which accounted for 20.16% of the total number of fungi strains screened, showed inhibitory activity against certain pathogens, of which most (18/25, 72%) had a wide spectrum, suggesting that endophytic fungi have a huge potential for synthesizing compounds with antibacterial activity. We further evaluated the activity of ethyl acetate extracts against veterinary and hospital multidrug-resistant pathogen isolates. Among these extracts, those from *P. sclerotigenum, D. kochmanii,* and *P. trachycarpicola* showed outstanding antibacterial and bactericidal activities against MRSA SMU3194, which may be related to the organic acids and flavonoids present in their related extracts. In addition, Shi et al. reported that genistein isolated from the endophytic fungus, *Penicillium brefeldianum* F4a, showed antioxidant, blood lipid-lowering, and antibacterial effects [34]. The antibacterial activity of phenyllactic acid is attributed to its ability to dissipate transmembrane potential and increase cell membrane permeability, resulting in the leakage of intracellular potassium ions. Phenyllactic acid alters cell morphology and bacterial adhesion by disrupting cell membrane integrity. After entering the cells, the DNA structure can be disrupted by phenyllactic acid, which also inhibits gene expression and could subsequently lead to bacterial disintegration [35].

Antibacterial compounds mainly act on bacterial cell walls, plasma membranes, proteins, and nucleic acid synthesis, inhibiting DNA replication and transcription to kill pathogenic bacteria [36]. Approximately 80% of bacterial infections are associated with biofilm formation. When compared to free bacteria, biofilms increase antibiotic resistance 10–1000-fold and are the main cause of drug resistance in bacteria [37]. Among the identified compounds, we focused on flavonoids, a diverse group of heterocyclic organic compounds. Their antibacterial activity is associated with the number of hydroxyl groups in the flavonoid aromatic ring and substitution positions [38]. Gong et al. reported that the main anti-gram-positive bacteria mechanism of flavonoid compounds was targeting the cell membrane of bacteria, causing cell membrane damage, and inhibiting the oxidative respiratory chain, as well as adenosine triphosphate synthesis [39]. The results of this study proved that the ethyl acetate extracts of *P. sclerotigenum, D. kochmanii,* and *P. trachycarpicola* showed anti-biofilm activity against test strains and caused significant damage to the biofilm matrix.

Since *P. sclerotigenum, D. kochmanii,* and *P. trachycarpicola* obtained from *A. adenophora* showed drug development potential, we further used the LC-MS to analyze the chemical components of their extracts, and some of the identified compounds were found to have antibacterial, antineoplastic, and blood lipid-lowering bioactivities based on published research. However, these compounds were obtained from slow-growing and/or rare and endangered plants rather than microorganisms. In the present study, *P. sclerotigenum*, *D. kochmanii*, and *P. trachycarpicola* synthesized rich, diverse, and bioactive secondary metabolites, such as phenolic acids, fatty acids, organic acids, and flavonoids. Organic acids and flavonoids are secondary metabolites with a broad spectrum of pharmacological activity [40]. For bacteriostatic activity, organic acids disrupt the outer membrane of bacteria, increase intracellular osmotic pressure, inhibit macromolecule synthesis, and cause the host to produce antibacterial peptides [41]. In some previous studies, antibacterial compounds such as 4-α-D-glucopyranosyl-1→4-β-L-rhamnopyranosyloxy)-benzyl thiocarboxamide and 21-acetoxycytochalasin were extracted from the cultures of *P. sclerotigenum* and *Diaporthe* sp. GDG-118 [42,43]. Similarly, two new isocoumarin compounds, pestaloisocoumarins A and B, isolated from the endophytic fungus *P. heterocornis,* showed potent cytotoxic and antibacterial activities [44]. These results showed that *P. sclerotigenum, D. kochmanii*, and *P. trachycarpicola* isolated from *A. adenophora* contain many bioactive compounds, representing a molecular source with potential pharmacological application value. Therefore, in the future, these bioactive substances can be easily obtained via fermentation rather than plant tissues since the diverse chemical components in endophytic fungi are easy to culture and show significant bioactivity [19]. In addition, physical chemistry and gene manipulation techniques can be used to increase the production of drug-specific secondary metabolites in fungal endophytes. In summary, endophytic fungi from *A. adenophora* are the potential source of novel antibiotics for treating both human and animal pathogen infections. We recommend that future studies focus on the chemical characterization and structural elucidation of these bioactive substances. Furthermore, the mechanisms of action of each bioactive substance in performing bactericidal activity can be identified, as well as several in vivo studies can be performed to validate the activity of these secondary metabolites.

## 4. Materials and Methods

### 4.1. Study Site and Collection of Plant Materials

The *A. adenophora* plant was collected from Mount Luoji, Liangshan Yi Autonomous Prefecture, Sichuan Province, on July 2020 (coordinates: 27°35′16′′ N and 102°24′29′′ E, altitude: 2531 m). The plant was collected from public lands or areas with low human interference and was identified by Prof. Chao Hu, Department of Botany, Sichuan Agricultural University. *A. adenophora* plant that showed exuberant growth and coverage of 90–100%, was sampled with the sampling interval >50 m [45]. Then the plant materials were collected using sterile scalpels. The roots, stems, and leaves were placed in sterile polyethylene bags and transported at a temperature of 4 °C to the Environmental Pollution and Animal Disease Laboratory of Sichuan Agricultural University.

### 4.2. Isolation of Endophytic Fungi

Plant samples were thoroughly cleaned under running tap water and air dried at room temperature for 4 h. Furthermore, the procedure for surface disinfectant by Zhao et al., with slight modifications to avoid the effects of epiphytic microorganisms, was followed [46]. Plant samples (roots, stems, and leaves) were soaked in 75% ethanol (*v*/*v*) for 2–5 min, 0.5% sodium hypochlorite (*v*/*v*) for 2–3 min, 75% ethanol for 0.5–1 min, and washed 3 times with sterile water and dried using sterile filter paper. After samples were cut into 0.5 cm^2^ slices using sterile scissors and transferred onto PDA petri dishes containing 100 µg/L ampicillin and kanamycin sulfate (Supplementary Figure S1). The last wash solution was inoculated on potato dextrose agar (PDA) petri dishes and cultured for 3 days at 25 °C to evaluate the results of surface disinfection of the samples. The petri dishes were sealed with parafilm and cultured at 25 °C for 7–10 days. Fungal colonies were inoculated onto fresh PDA petri dishes. Finally, the purified fungi were stored at −80 °C in a 20% glycerol solution at the Environmental Pollution and Animal Disease Laboratory of Sichuan Agricultural University.

### 4.3. Identification of Endophytic Fungi

The endophytic fungi were identified using their morphology and DNA sequence data. The morphological characterization was performed through the observation of four parameters: the colonies’ color of the upper surface, texture (fluffy/submissive), size of conidia, and mycelium length [47]. The morphological data of the isolates collected in the present study were obtained from sporulating pure cultures grown on PDA in the dark at 25 °C. For colony morphology determination, endophytic fungi were cultured on PDA media at 25 °C for 7 days. The colonies’ morphology and spores were observed and photographed using an Olympus BX53 microscope and a MicroPublisher 5.0 RTV digital camera (Olympus Corp., Tokyo, Japan). For mycelium observation, mycelial discs (5 mm) were removed from 7 day-old PDA plates and placed in the center of a slide. The slide was then stained with methylene blue and observed and photographed under an optical microscope (Nikon DS-Ril-U2, Tokyo, Japan). The morphological results were recorded and compared with knowledge from published literature for preliminary characterization, which was further investigated and verified by the DNA sequence shown below [48,49,50,51,52,53,54].

The cetyl trimethyl ammonium bromide method was used to extract fungal genomic DNA according to the instructions of the Omega fungal genomic DNA extraction kit (Omega Bio-Tek, Norcross, GA, USA). DNA concentration was quantitated (>100 ng/µL in a volume of 25 µL) using a NanoDrop One/OneC microvolume UV-Vis spectrophotometer (Thermo Fisher Scientific, Waltham, MA, USA). Subsequently, universal primers, ITS4 (5′-TCCTCCGCTTATTGATATGC-3′) and ITS5 (5′-GGAAGTAAAAGTCGTAACAAGG-3′), were used to amplify the 5.8 S ITS region in genomic DNA. The amplification reaction system and amplification conditions were the same as previously described [55]. Furthermore, 1% (*w*/*v*) agarose gel electrophoresis was used to analyze 5 µL of PCR product before the PCR product was sent to Sangon Biotech Co. Ltd. (Shanghai, China) for purification and sequencing. The amplified ITS (ITS4-5.8 S-ITS5) sequences and existing species sequences in GenBank were analyzed using Blast (http://www.ncbi.nlm.nih.gov/). The ITS sequences obtained in this study were submitted to NCBI GenBank, with accession numbers MZ047481-MZ047583 and MZ066737-MZ066757.

Four important parameters, Species abundance index (*S*), Margalef index (*D*^/^), Shannon-Wiener index (*H*^/^), and Simpson’s diversity index (*D*), were used to evaluate the diversity of endophytic fungi in *A. adenophora* [56]. The formulas were as follows:

*S = ni/N* (1)

*D^/^= (S − 1)/log_2_N* (2)

*H^/^= −∑Pi log_2_Pi* (3)

*D = 1 − ∑Pi^2^* (4)

Note: *ni* represents the number of isolates of the same species, *Pi* is the percentage of strains isolated from an endophytic fungal taxon in the total number of strains, and *N* represents the total number of endophytic fungi strains.

### 4.4. Antibacterial Activity Screening

#### 4.4.1. Agar Well Diffusion

The antibacterial activity of endophytic fungi was determined using agar well diffusion [57]. Five types of antibiotic-sensitive bacteria (*Escherichia coli* O157:H7 CICC21530, *Salmonella enteritidis* CICC24119, *Salmonella paratyphi* B CICC10437, *Staphylococcus aureus* CPCC140594, and *Streptococcus agalactiae* ATCC13813) were used as test microorganisms. Each strain of endophytic fungi was cultured on PDA at 25 °C for 7 days. Then, fungal plugs (6 mm in diameter) were excised and inoculated on potato dextrose broth (PDB) culture medium. For 14 days, all fungal strains were cultured in a shaking incubator at 25 °C and 150 rpm. The culture medum was filtered to isolate the mycelium and fermentation broth. The fermentation broth was concentrated using a rotary evaporator at 55 °C before being centrifuged at 5000 rpm for 10 min. The concentrated fermentation broth was then filtered using a water system filter to remove fungi. A double-layered plate was created in petri dishes, with water agar as the bottom layer and 1 × 10^4–6^ CFU/mL bacteria-containing MHA as the top layer. After the bacteria-containing layer had solidified, an Oxford cup was placed in it, and 20 μL of fermentation broth filtrate was added. After incubation at 37 °C for 24 h, electronic digital calipers were used to measure the inhibition diameter zone to evaluate antibacterial activity. All experiments were repeated three times.

#### 4.4.2. Preparation of EtOAc Extracts

Isolates that passed the preliminary agar well diffusion screening were cultured on PDA for 7 days before being inoculated into the 500 mL conical flasks containing 200 mL culture medium. All the fungal strains were cultured at 25 °C in a shaking incubator of 150 rpm for 3 days to obtain pre-inoculum. Afterwards, 20 mL of pre-inoculum (5 bottles for every fermentation strain) was added to a 400 mL PDB culture broth, and cultured at 25 °C static conditions for 21 days. Ethyl acetate (3 × 400 mL) was used to extract the culture filtrate. The organic phase of the culture filtrate was placed in a rotary evaporator and a 40 °C water bath, in order to remove organic solvents. The mycelia were dried at 60 °C and pulverized. A total of 300 mL of 70% methanol was added, and ultrasonic extraction was performed for 1 h (3 extractions in total). The leachate was vacuum-concentrated to obtain the crude metabolic extract of mycelia. The combined filtrates with crude organic extracts were combined and lyophilized [58]. Using 10% DMSO, the crude extract was diluted to 8 mg/mL. A 0.22 μm organic filter was used for filter sterilization. The filtrates were used to determine the minimum inhibitory concentration (MIC) and minimum bactericidal concentration.

### 4.5. Measurement of MIC and Minimum Bactericidal Concentration

Following the recommendations approved by the US National Committee for Clinical Laboratory Standards, the modified broth microdilution was used to measure the MIC of ethyl acetate extracts against four types of veterinary and clinical multidrug-resistant pathogen isolates: gram-negative bacteria (*E. coli* SMU1710 and *Salmonella* SMU3256) and gram-positive bacteria (MRSA SMU3194 and *S. agalactiae* SMU5052) [59,60]. First, the pathogen was inoculated in Muller-Hinton broth (MHB) and cultured overnight at 37 °C. The culture density was adjusted from 600 nM to 0.6 optical density. Continuous microdilution was used to dilute the ethyl acetate extract in MHB broth (0–4 mg/mL). Then, the test strain (10 μL) and MHB broth (100 μL) were separately inoculated in wells with different concentrations of samples. The final concentration range was 0–2 mg/mL. MHB broth was used as the negative control, and ceftiofur sodium and vancomycin were used as the positive controls for gram-negative and gram-positive bacteria, respectively (50 µg/L). The 96-well plates were incubated at 37 °C for 4 h, and 0.1 mg/mL of MTT (20 μL) was added to each well and incubated for another 4 h. A color change from light purple to purple indicated no bacterial growth and was marked as negative. The minimum extract concentration that inhibited pathogenic bacterial growth was recorded as the MIC. The MIC results were used to confirm the concentration at which there was no visible growth. In determining this, 50 μL of culture medium was transferred to a Mueller-Hinton agar (MHA) plate before culturing at 37 °C for 24 h. The absence of bacterial growth on the MHA surface at the minimum extract concentration was defined as the minimum bactericidal concentration (MBC). All experiments were repeated three time [61].

### 4.6. Fluorescence Microscopy

Acridine orange fluorescence stain was used to observe the biofilms of 2MIC fungal ethyl acetate extract-treated *E. coli* SMU1710, *Salmonella* SMU3256, and MRSA SMU3194 [37]. Further, the biofilms were grown on cell slides for 48 h, and the PBS buffer was used to gently wash the slides twice to remove surface nonadherent bacteria. To fix biofilms, the slides were naturally dried for 10 min before being immersed in 10 μL of fixing solution (the fixing solution consists of glacial acetic acid, chloroform, and absolute ethanol, respectively 1:3:6). Acridine orange (0.1% *w*/*v*, dissolved in 1× PBS) was used to stain biofilms formed on cell slides. Finally, 10 μL of anti-fluorescence quenching mounting solution was added onto the biofilms, and the slides were placed under a BX53 upright fluorescence microscope (Olympus, Tokyo, Japan) to observe biofilms.

### 4.7. Liquid Chromatography–Mass Spectrometry Analysis of the Chemical Composition of Ethyl Acetate Extracts

The bioactive compounds in the endophytic fungi ethyl acetate extracts were analyzed using UHPLC-HRMS (Thermo, Vanquish UHPLC; Thermo, Orbitrap Q ExactiveTMHF-X). The separation of compounds was achieved on a Hypersil Gold column C18 (2.1 × 100 mm i.d) with a bead size of 1.9 µm. The eluents for the positive polarity mode were eluent A (0.1% FA in water) and eluent B (methanol), and the flow rate was 0.2 mL min^−1^. The chromatography gradient-elution program was as follows: 2% B, 1.5 min; 2–100% B, 3 min; 100% B, 10 min; 100–2% B, 10.1 min; and 2% B, 12 min. Positive/negative polarity mode ionization (electrospray voltage: 3.5 KV; sheath gas flow rate: 35 psi; auxiliary gas flow rate: 10 L/min; ion transmission tube temperature: 320 °C; iontophoresis radiofrequency level: 60; auxiliary gas heater temperature: 350 °C) MS/MS secondary scan (data-dependent scan) with a scanning range of 100–1500 m/z was used for mass spectrometry. Compound Discoverer 3.1 (Thermo Scientific, Waltham, MA, USA) analysis was used to obtain the mass spectra, whereas the mzCloud, mzVaulat, and Mass List databases were used for nontargeted detection of secondary metabolites.

### 4.8. Statistical Analyses

All experimental data were expressed as mean ± standard deviation of three independent experiments. SPSS 26.0 software (IBM, Armonk, NY, USA) was used for one-way analysis of variance (ANOVA) and Duncan’s multiple range test, where *p <* 0.05 indicated that the differences between treatments were statistically significant.

## 5. Conclusions

Taken together, the results obtained in this study showed that extracts from *P. sclerotigenum, D. kochmanii,* and *P. trachycarpicola* isolated from *A. adenophora* showed potential antibacterial activity against veterinary and hospital multidrug-resistant *Staphylococcus aureus*. We therefore speculated that these antibacterial activities were associated with organic acids and flavonoids present in the crude extract of these microbes. Therefore, there is a need for more detailed subsequent studies on the application of these endophytic fungi in antibiotic drug research and development.

## Figures and Tables

**Table 1:** Molecular identification of culturable endophytic fungi in *A. adenophora* based on GenBank database basic local alignment search tool (BLAST).

No.	Closest Species in ITS Gene Sequences Database	Strain Number (DNA Sequence Data)	Identity (%)	N	IF
1	*Alternaria alternata* isolate 1 (MH368103)	DCL02, DCL03, DCL11, DCL12, DCL13, DCL15, DCL16, DCL17, DCL18, DCL24,DCL26, DCL34, DCL37, DCL39,DCL46, DCS01, DCS04, DCS06,DCS11, DCS13, DCS15, DCS16, DCS17, DCS18, DCS28, DCS29, DCS32, DCR23	99.47	28	22.58
2	*Alternaria dauci* strain SM19 (MZ314740)	DCS03	99.46	1	0.81
3	*Alternaria tenuissima voucher* HGUP191067 (MZ541977)	DCL14, DCL21, DCL25,DCL32, DCS05	100.00	5	4.03
4	*Aspergillus flavus* isolate AMS_3 (MW522551)	DCL04, DCL10	99.49	2	1.61
5	*Aporospora terricola* strain (MW961422)	DCL01	100.00	1	0.81
6	*Cercospora* sp. IPBCC 13.1012 (KC776152.1)	DCL22, DCR24	99.43	2	1.61
7	*Botrytis cinerea* isolate ET 63 (MH992149)	DCL50, DCL54	99.62	2	1.61
8	*Diaporthe novem* strain MLT18 (MH299960)	DCL27, DCL40, DCL43, DCR08, DCR35	99.47	5	4.03
9	*Pestalotiopsis trachycarpicola voucher* HGUP194013 (MZ724924)	DCL44, DCL47, DCL48, DCR34	99.67	4	3.23
10	*Trametes versicolor* isolate Au-I-1.1 (MF475935)	DCL31	99.34	1	0.81
11	*Trametes hirsuta* isolate HH1 (MF377430)	DCL33	99.84	1	0.81
12	*Ampelomyces* sp. isolate X13 (KJ958371)	DCL28	99.81	1	0.81
13	*Phoma* sp. strain CN1 (ON025541)	DCS07	99.62	1	0.81
14	*Phomopsis* sp. FH-2012b isolate cgyg1 (JQ954648)	DCL38, DCR04	98.95	2	1.61
15	*Botrytis fabae* strain DH-6 (MN589851)	DCL51	99.43	1	0.81
16	*Cladosporium* sp. WSN6 (KC178629)	DCL23, DCS14, DCS23, DCS24, DCR05,DCR21	100.00	6	4.84
17	*Cladosporium oxysporum* ALSHB3 (KU561865)	DCR19	100.00	1	0.81
18	*Cladosporium perangustum* isolate A743 (KU529857)	DCS21	99.81	1	0.81
19	*Colletotrichum* sp. JT2 (KC507287.1)	DCL30	99.65	1	0.81
20	*Colletotrichum gloeosporioides* strain GZBY01 (KM044004)	DCL36, DCL49	100.00	2	1.61
21	*Colletotrichum godetiae* strain 435E (MZ078527.1)	DCL55	99.82	1	0.81
22	*Colletotrichum liriopes* isolate SJM3-2 (MN589679)	DCS20	99.47	1	0.81
23	*Colletotrichum siamense* isolate GQH57(MN296041.1)	DCL35, DCL52	99.65	2	1.61
24	*Fusarium oxysporum* clone SF_72 (MT529348.1)	DCL07, DCL53, DCS22, DCR06, DCR29, DCR30, DCR31	100.00	7	5.65
25	*Fusarium acuminatum* isolate Kt6.1 (MN489462)	DCR20	99.46	1	0.81
26	*Fusarium graminearum* isolate TD2 (MT228970)	DCL19	99.81	1	0.81
27	*Fusarium kyushuense* isolate G797 (MK247795)	DCR07	100.00	1	0.81
28	*Fusarium solani* strain K. L. Chen L035 (KX034335)	DCR09, DCR12, DCR15, DCR16	99.82	4	3.23
29	*Fusarium verticillioides* strain JINF002 (KX196811)	DCS10	99.44	1	0.81
30	*Trichoderma gamsii* strain ICC080 (GQ351597)	DCR22	99.33	1	0.81
31	*Trichoderma longibrachiatum* isolate BM10 (MK910065)	DCL05	99.37	1	0.81
32	*Trichothecium roseum* isolate JZB57004 (MW440515)	DCS25	99.83	1	0.81 2.42
33	*Trichoderma tomentosum* DAOM 229898 (AY605737)	DCS08,DCS09,DCS30,DCR28	99.67	4	3.23
34	*Trichoderma sulphureum* clone SF_102 (MT529378)	DCS27, DCS31	100.00	2	1.61
35	*Brunneomyces brunnescens* isolate NTOU5435 (MN592899)	DCL41	98.56	1	0.81
36	*Paecilomyces variotii* isolate RS3-S2-27 (MN547409)	DCR18	99.65	1	0.81
37	*Didymella* sp. SS14 (KC507292.1)	DCL08, DCR02, DCR11 DCR02 DCR11	100.00	3	2.42
38	*Didymella sinensis* isolate BY42 (MH257405)	DCL42	99.05	1	0.81
39	*Diaporthe ambigua* isolate UT15JD (MF319487)	DCL56, DCR27, DCR36	99.47	3	2.42
40	*Diaporthe actinidiae* strain JL2 (KT163360)	DCL29	100.00	1	0.81
41	*Diaporthe kochmanii* voucher HGUP193007 (MZ724751)	DCL09	99.82	1	0.81
42	*Gregarithecium curvisporum* HHUF 30134 (NR154049)	DCR17	92.40	1	0.81
43	*Mucor fragilis* isolate MZC-1 (MN069560)	DCR10, DCR14	99.83	2	1.61
44	*Nigrospora sphaerica* strain AL2 (MT466514)	DCR33	99.45	1	0.81
45	*Penicillium commune* isolate K19(MK179259)	DCL20,DCS12, DCR01	99.47	3	2.42
46	*Penicillium concentricum* isolate C3 (EU551180)	DCR03	99.47	1	0.81
47	*Penicillium ochrochloron* strain KD-F1 (MK720828)	DCS26, DCR25	99.65	2	0.81
48	*Penicillium olsonii* strain KG 08/11/15 #2(MG252481)	DCS19	99.47	1	0.81
49	*Penicillium* sp. 14 BRO-2013 (KF367512.1)	DCR26	99.65	1	0.81
50	*Penicillium sclerotigenum* isolate INF71(MZ227345)	DCL06	100.00	1	0.81
51	*Plectosphaerella* sp. isolate F49 (MT771317)	DCR13	98.79	1	0.81
52	*Stagonosporopsis cucurbitacearum* strain CAP14C (JQ936326.1)	DCL45, DCS02, DCR32	99.81	3	2.42
Total		124			100
Species Richness (*S*)		52			
Margalef index (*D^/^*)		7.3337			
Shannon-Wiener index (*H^/^*)		3.6745			
Simpson’ s diversity index(*D*)		0.9304			

**Table 2:** Taxa of culturable endophytic fungi isolates from *A. adenophora*.

No.	Phylum	Class	Order	Family	Genus	N
1	Ascomycota	Dothideomycetes	Pleosporales	Pleosporineae	*Alternaria*	34
2				Didymellaceae	*Didymella*	4
3					*Phoma*	1
4				Leptosphaeriaceae	*Ampelomyces*	1
5				Dictyosporiaceae	*Gregarithecium*	1
6			Mycosphaerellales	Mycosphaerellaceae	*Cercospora*	2
7					*Stagonosporopsis*	3
8		Eurotiomycetes	Capnodiales	Cladosporiaceae	*Cladosporium*	8
9			Eurotiales	Aspergillaceae	*Aspergillus*	2
10					*Penicillium*	9
11				Thermoascaceae	*Paecilomyces*	1
12		Sordariomycetes	Diaporthales	Diaporthaceae	*Diaporthe*	10
13				Valsaceae	*Phomopsis*	2
14			Hypocreales	Nectriaceae	*Fusarium*	15
15				Hypocreaceae	*Trichoderma*	8
16					*Trichothecium*	1
17			Glomerellales	Plectosphaerellaceae	*Brunneomyces*	1
18					*Plectosphaerella*	1
19			Xylariales	Sporocadaceae	*Pestalotiopsis*	4
20				Apiosporaceae	*Nigrospora*	1
21			Glomerellales	Glomerellaceae	*Colletotrichum*	7
22		Leotiomycetes	Helotiales	Sclerotiniaceae	*Botrytis*	3
23		Pezizomycotina			*Aporospora*	1
24	Basidiomycota	Agaricomycetes	Polyporales	Polyporaceae	*Trametes*	2
25	Mucoromycota	Mucoromycetes	Mucorales	Mucoraceae	*Mucor*	2
Total	3	7	12	19	25	124

**Table 3:** Antibacterial activity of endophytic fungi isolated from *A. adenophora*.

Endophytic Fungi		Pathogens Inhibitory Zone Measurement (mm)				
		*E. coli* CICC21530	*S. enteritidis* CICC24119	*S. paratyphi* CICC10437	*S. agalactiae* ATCC13813	*S. aureus* CPCC140594
DCL03	*Alternaria alternata*	16.19 ± 0.48 ^cd^	0.00 ± 0.00 ^a^	0.00 ± 0.00 ^a^	0.00 ± 0.00 ^a^	10.88 ± 0.58 ^fg^
DCL06	*Penicillium sclerotigenum*	20.62 ± 0.74 ^fg^	19.28 ± 0.12 ^ef^	17.35 ± 1.22 ^cd^	18.69 ± 0.44 ^fg^	8.89 ± 0.73 ^de^
DCL09	*Diaporthe kochmanii*	19.05 ± 1.35 ^ef^	18.65 ± 0.34 ^de^	17.86 ± 0.47 ^cd^	19.09 ± 0.66 ^fg^	8.05 ± 0.57 ^cd^
DCL12	*Alternaria alternata*	0.00 ± 0.00 ^a^	14.80 ± 0.53 ^b^	14.44 ± 0.97 ^b^	8.14 ± 0.44 ^b^	7.18 ± 0.78 ^c^
DCL14	*Alternaria tenuissima*	21.95 ± 1.44 ^g^	20.31 ± 0.19 ^fg^	0.00 ± 0.00 ^a^	19.92 ± 0.23 ^g^	0.00 ± 0.00 ^a^
DCL16	*Alternaria alternata*	0.00 ± 0.00 ^a^	0.00 ± 0.00 ^a^	0.00 ± 0.00 ^a^	0.00 ± 0.00 ^a^	9.98 ± 0.81 ^ef^
DCL18	*Alternaria alternata*	0.00 ± 0.00 ^a^	21.37 ± 1.02 ^g^	0.00 ± 0.00 ^a^	17.65 ± 1.30 ^def^	12.12 ± 0.91 ^g^
DCL24	*Alternaria alternata*	17.79 ± 1.79 ^cde^	0.00 ± 0.00 ^a^	0.00 ± 0.00 ^a^	14.07 ± 0.76 ^c^	0.00 ± 0.00 ^a^
DCL28	*Ampelomyces* sp.	17.04 ± 1.28 ^cde^	0.00 ± 0.00 ^a^	16.73 ± 1.08 ^cd^	16.64 ± 0.47 ^d^	8.93 ± 0.79 ^de^
DCL31	*Trametes versicolor*	0.00 ± 0.00 ^a^	17.49 ± 0.36 ^cd^	0.00 ± 0.00 ^a^	17.49 ± 0.93 ^def^	0.00 ± 0.00 ^a^
DCL36	*Colletotrichum gloeosporioides*	15.55 ± 0.60 ^c^	17.57 ± 1.24 ^cd^	0.00 ± 0.00 ^a^	17.50 ± 0.74 ^def^	10.38 ± 0.63 ^ef^
DCL44	*Pestalotiopsis trachycarpicola*	18.06 ± 1.01 ^de^	19.95 ± 0.51 ^ef^	16.43 ± 1.64 ^c^	16.82 ± 0.97 ^d^	10.33 ± 0.63 ^ef^
DCL48	*Pestalotiopsis trachycarpicola*	16.56 ± 0.57 ^cd^	16.21 ± 1.05 ^bc^	14.54 ± 0.71 ^b^	0.00 ± 0.00 ^a^	7.57 ± 0.32 ^cd^
DCL51	*Botrytis fabae*	12.96 ± 0.82 ^b^	0.00 ± 0.00 ^a^	14.84 ± 0.42 ^b^	0.00 ± 0.00 ^a^	7.96 ± 0.55 ^cd^
DCS07	*Phoma* sp.	0.00 ± 0.00 ^a^	0.00 ± 0.00 ^a^	0.00 ± 0.00 ^a^	0.00 ± 0.00 ^a^	7.89 ± 0.24 ^cd^
DCS23	*Cladosporium* sp.	0.00 ± 0.00 ^a^	0.00 ± 0.00 ^a^	0.00 ± 0.00 ^a^	19.85 ± 1.25 ^g^	0.00 ± 0.00 ^a^
DCS28	*Alternaria alternata*	0.00 ± 0.00 ^a^	0.00 ± 0.00 ^a^	0.00 ± 0.00 ^a^	19.04 ± 0.48 ^fg^	0.00 ± 0.00 ^a^
DCS30	*Trichoderma tomentosum*	0.00 ± 0.00 ^a^	0.00 ± 0.00 ^a^	0.00 ± 0.00 ^a^	19.27 ± 0.55 ^c^	7.01 ± 1.06 ^fg^
DCR04	*Phomopsis* sp.	0.00 ± 0.00 ^a^	19.73 ± 0.91 ^ef^	17.47 ± 1.07 ^cd^	19.42 ± 0.54 ^g^	10.94 ± 0.71 ^fg^
DCR09	*Fusarium solani*	19.02 ± 0.42 ^ef^	0.00 ± 0.00 ^a^	0.00 ± 0.00 ^a^	0.00 ± 0.00 ^a^	10.39 ± 0.41 ^ef^
DCR12	*Fusarium solani*	17.97 ± 1.47 ^de^	19.54 ±0.88 ^ef^	16.23 ± 0.20 ^c^	17.62 ± 0.07 ^def^	5.58 ± 0.15 ^b^
DCR16	*Fusarium solani*	17.76 ± 1.48 ^cde^	15.52 ± 0.80 ^b^	0.00 ± 0.00 ^a^	17.18 ± 1.19 ^de^	10.13 ± 0.81 ^ef^
DCR19	*Cladosporium oxysporum*	0.00 ± 0.00 ^a^	19.19 ± 0.93 ^df^	18.21 ± 0.60 ^d^	0.00 ± 0.00 ^a^	0.00 ± 0.00 ^a^
DCR25	*Penicillium ochrochloron*	11.41 ± 0.97 ^b^	18.38 ± 0.33 ^de^	16.49 ± 0.46 ^c^	13.36 ± 1.36 ^c^	7.93 ± 0.93 ^cd^
DCR34	*Pestalotiopsis trachycarpicola*	0.00 ± 0.00 ^a^	0.00 ± 0.00 ^a^	0.00 ± 0.00 ^a^	19.98 ± 0.89 ^g^	11.99 ± 0.30 ^g^

CICC: China Center of Industrial Culture Collection, ATCC: American Type Culture Collection, CPCC: China Pharmaceutical Culture Collection. The results are expressed as mean ± SEM. The same superscript letter (a–g) means that there was no significant difference in the inhibition diameter zone of antibiotic-sensitive bacteria by different endophytic fungi (*p* > 0.05) (same row).

**Table 4:** Minimum inhibitory concentration and minimum bactericidal concentration (mg/mL) of ethyl acetate extracts from endophytic fungi isolated from *A. adenophora* against veterinary and clinical multidrug-resistant pathogen isolates.

Crude Extracts of the Fungal Endophytes	Gram-Negative Bacteria				Gram-Positive Bacteria			
	*E. coli* SMU1710		*Salmonella* SMU3256		MRSA SMU3194		*S.agalactiae* SMU5052	
	MIC	MBC	MIC	MBC	MIC	MBC	MIC	MBC
*P. sclerotigenum*	0.5	2	2	nd	0.5	2	0.5	nd
*D. kochmanii*	2	nd	1	2	0.5	1	2	nd
*P. trachycarpicola*	1	2	0.5	2	1	2	2	nd
*F. solani*	nd	nd	nd	nd	2	nd	nd	nd
*P. ochrochloron*	nd	nd	1	nd	nd	nd	2	nd

Veterinary hospital strains: SMU (Southwest Minzu University), MRSA: Methicillin-resistant *Staphylococcus aureus*, nd: not detected (result higher 2 mg/mL).

**Table 5:** Chemical composition of ethyl acetate extracts from endophytic fungi identified via liquid chromatography–tandem mass spectrometry.

S/N	Name of Identified Compound	Adducts	Molecular Formula	RT (Min)	M/Z	Endophytic Fungi (µmol/g)		
						*P. sclerotigenum*	*D. kochmanii*	*P. trachycarpicola*
1	D-(+)-Mannose	[M-H]^−^	C_6_H_12_O_6_	1.419	179.056	2.29	0.36	1.85
2	Sucrose	[M-H]^−^	C_12_H_22_O_11_	1.495	341.109	0.64	1.50	1.09
3	D-(−)-Fructose	[M-H]^−^	C_6_H_12_O_6_	1.560	179.056	0.75	0.18	1.93
4	DL-Malic acid	[M-H]^−^	C_4_ H_6_O_5_	1.579	133.014	4.61	nd	3.39
5	trans-Aconitic acid	[M-H]^−^	C_6_H_6_O_6_	1.598	173.009	0.38	nd	0.01
6	Pipecolic acid	[M-H]^−^	C_6_H_11_NO_2_	1.743	130.086	0.83	1.29	0.02
7	D-(+)-Malic acid	[M-H]^−^	C_4_H_6_O_5_	1.767	133.014	1.02	nd	0.13
8	D-α-Hydroxyglutaric acid	[M-H]^−^	C_5_H_8_O_5_	1.999	147.030	1.27	0.01	nd
9	Citric acid	[M-H]^−^	C_6_ H_8_O_7_	2.006	191.020	3.60	5.61	21.14
10	Glutaconic acid	[M-H]^−^	C_5_H_6_O_4_	2.112	129.019	nd	1.91	nd
11	Coumarin	[M-H]^−^	C_9_H_6_O_2_	2.357	147.044	0.01	0.14	0.39
12	Fumaric acid	[M-H]^−^	C_4_H_4_O_4_	2.531	115.003	nd	0.01	2.51
13	Succinic acid	[M-H]^−^	C_4_H_6_O_4_	2.696	117.019	0.58	nd	1.34
14	4-Hydroxybutyric acid	[M-H]^−^	C_4_H_8_O_3_	3.756	103.040	nd	nd	0.26
15	2′-Deoxyinosine	[M-H]^−^	C_10_H_12_N_4_O	4.094	251.078	0.80	nd	0.24
16	Homogentisic acid	[M-H]^−^	C_8_H_8_O_4_	4.846	167.035	1.71	1.05	nd
17	2-Furoic acid	[M-H]^−^	C_5_H_4_O_3_	4.894	111.008	0.76	nd	nd
18	Pantothenic acid	[M-H]^−^	C_9_H_17_NO_5_	5.111	218.103	1.12	nd	2.32
19	Caffeic acid	[M-H]^−^	C_9_H_8_O_4_	5.167	179.035	0.32	4.38	0.71
20	Salicylic acid	[M-H]^−^	C_7_H_6_O_3_	5.413	137.024	0.32	0.19	0.34
21	Taxifolin	[M-H]^−^	C_15_H_12_O_7_	5.555	303.051	1.15	nd	nd
22	Benzoic acid	[M-H]^−^	C_7_H_6_O_2_	5.561	121.029	0.22	0.01	0.17
23	2,3-Dihydroxybenzoic acid	[M-H]^−^	C_7_H_6_O_4_	5.586	153.019	0.63	nd	0.11
24	3-Coumaric acid	[M-H]^−^	C_9_H_8_O_3_	5.649	163.040	nd	0.59	1.71
25	N-Acetylvaline	[M-H]^−^	C_7_H_13_NO_3_	5.663	158.082	0.37	1.07	0.48
26	Phenylacetylglycine	[M-H]^−^	C_10_H_11_NO_3_	5.685	192.067	3.39	0.92	0.50
27	Suberic acid	[M-H]^−^	C_8_H_14_O_4_	5.719	173.082	0.03	0.22	2.61
28	N-Acetyl-L-phenylalanine	[M-H]^−^	C_11_H_13_NO_3_	5.730	206.082	nd	nd	3.21
29	2-Hydroxycaproic acid	[M-H]^−^	C_6_ H_12_O_3_	5.737	131.071	1.66	nd	nd
30	Phenyllactic acid	[M-H]^−^	C_9_H_10_O_3_	5.754	165.056	1.20	9.59	nd
31	Glycitein	[M-H]^−^	C_16_H_12_O_5_	5.888	284.069	0.93	1.17	0.28
32	Azelaic acid	[M-H]^−^	C_9_H_16_O_4_	5.908	187.098	0.30	0.81	1.37
33	Genistein	[M-H]^−^	C_15_H_10_O_5_	6.043	269.045	2.85	nd	4.70
34	Nootkatone	[M-H]^−^	C_15_H_22_O	6.303	219.174	1.67	nd	nd
35	Dulcitol	[M-H]^−^	C_6_H_14_O_6_	7.527	181.072	0.11	0.04	0.29
36	1,5-Anhydro-D-glucitol	[M-H]^−^	C_6_H_12_O_5_	8.058	163.061	2.66	nd	0.13
37	Xylitol	[M-H]^−^	C_5_H_12_O_5_	8.707	151.061	1.10	0.14	0.19
38	Isorhamnetin	[M-H]^−^	C_16_H_12_O_7_	10.283	315.051	6.21	0.12	0.01

Note: RT, Retention Time. M/Z, Mass-to-Charge Ratio. nd, not detect.

## Data Availability

The data presented in this study are available on request from the corresponding author.

## Supplementary Materials

The following supporting information can be downloaded at: https://www.mdpi.com/article/10.3390/plants12030650/s1. Figure S1: Endophytic fungi were isolated from fresh leaves of *A. adenophora* by surface disinfection and tissue separation; Figure S2: Agarose electrophoresis of amplified ITS region of endophytic fungi; Figure S3: Total ion chromatogram of Ethyl Acetate extract of endophytic fungi; Table S1: The identification results of cultivable endophytic fungi from *A. adenophora*.

## Abbreviations

BLAST	Basic Local Alignment Search Tool
NCBI	North Carolina Banking Institute
ITS	Internal transcribed spacer
DNA	Deoxyribonucleic Acid
PCR	Polymerase chain reaction
SSA	Similarity threshold
N	The number of isolates
IF	Isolation frequency
