# Supplementary material for: Endophytic Fungi Isolated from Ageratina adenophora Exhibits Potential Antimicrobial Activity against Multidrug-Resistant Staphylococcus aureus
Source: Plants (Basel). 2023 Feb 1;12(3):650. doi: 10.3390/plants12030650 (PMC9920656; doi:10.3390/plants12030650)
Supplement: Supplementary file 1 [file plants-12-00650-s001.zip › plants-2122648-supplementary.pdf]

plants

Article

Endophytic Fungi Isolated from *Ageratina adenophora* Exhibits Potential Antimicrobial Activity against Multidrug-Resistant *Staphylococcus aureus*

Juan Wen [1,†], Samuel Kumi Okyere [1,2,†], Jianchen Wang [1], Ruya Huang [1], Ya Wang [1], Lin Liu [3], Xiang Nong [4] and Yanchun Hu [1,5,*]

[1] Key Laboratory of Animal Disease and Human Health of Sichuan Province, Sichuan Agricultural University, Chengdu 611130, China; juanwen881010@163.com (J.W., Juan Wen); samuel20okyere@gmail.com (S.K.O.); wangjianchen01@163.com (J.W., Jianchen Wang); m18064929110@163.com (R.H.); wangyayang@126.com (Y.W.)
[2] Department of Pharmaceutical Sciences, School of Medicine, Wayne State University, Detroit, MI 48201, USA
[3] College of Grassland Science and Technology, Sichuan Agricultural University, Chengdu 611130, China; liulinsky@126.com
[4] College of Life Science, Leshan Normal University, Leshan 614000, China; nongx2008@163.com
[5] New Ruipeng Pet Healthcare Group Co., Ltd., Shenzhen 518000, China
* Correspondence: hychun114@163.com, yanchunhu@sicau.edu.cn; Tel.: +86-2886291162
† These authors contributed equally to this work.

Abstract: Multidrug-resistant bacteria such as *Staphylococcus aureus* (MRSA) cause infections that are difficult to treat globally, even with current available antibiotics. Therefore, there is an urgent need to search for novel antibiotics to tackle this problem. Endophytes are a potential source of novel bioactive compounds; however, the harnessing of novel pharmacological compounds from endophytes is infinite. Therefore, this study was designed to identify endophytic fungi (from *Ageratina adenophora*) with antibacterial activity against multidrug-resistant bacteria. Using fungal morphology and ITS-rDNA, endophytic fungi with antibacterial activities were isolated from *A. adenophora*. The results of the ITS rDNA sequence analysis showed that a total of 124 morphotype strains were identified. In addition, Species richness (*S*, 52), Margalef index (D', 7.3337), Shannon–Wiener index (H',3.6745), and Simpson's diversity index (*D*, 0.9304) showed that *A. adenophora* have abundant endophytic fungi resources. Furthermore, the results of the agar well diffusion showed that the *Penicillium sclerotigenum*, *Diaporthe kochmanii*, and *Pestalotiopsis trachycarpicola* endophytic fungi's ethyl acetate extracts showed moderate antibacterial and bactericidal activities, against methicillin-resistant *Staphylococcus aureus* (MRSA) SMU3194, with a MIC of 0.5–1 mg/mL and a MBC of 1–2 mg/mL. In summary, *A. adenophora* contains endophytic fungi resources that can be pharmacologically utilized, especially as antibacterial drugs.

Keywords: *Ageratina adenophora*; antibacterial activity; endophytic fungi; LC-MS analysis; secondary metabolites

Supplementary Materials

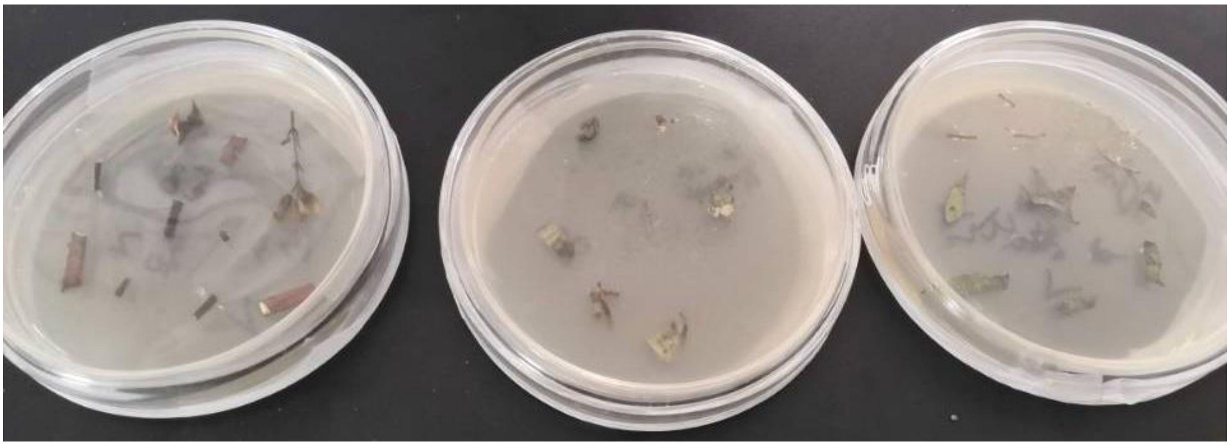

Figure S1. Surface-sterilize of *Ageratina adenophora.*

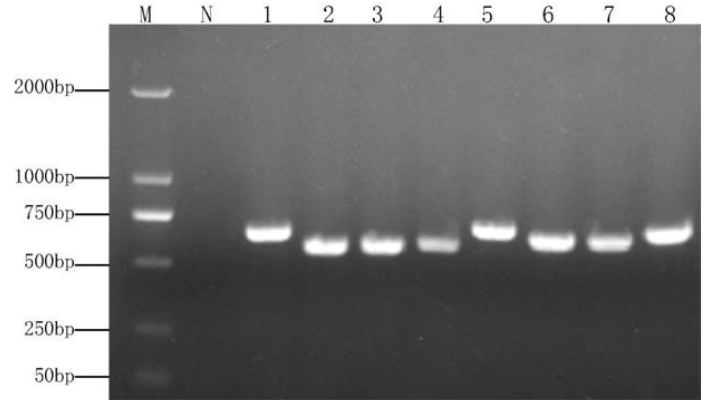

Figure S2: ITS-PCR Amplification Electrophoresis Map of Some Endophytic Fungi from *Ageratina adenophorum.*

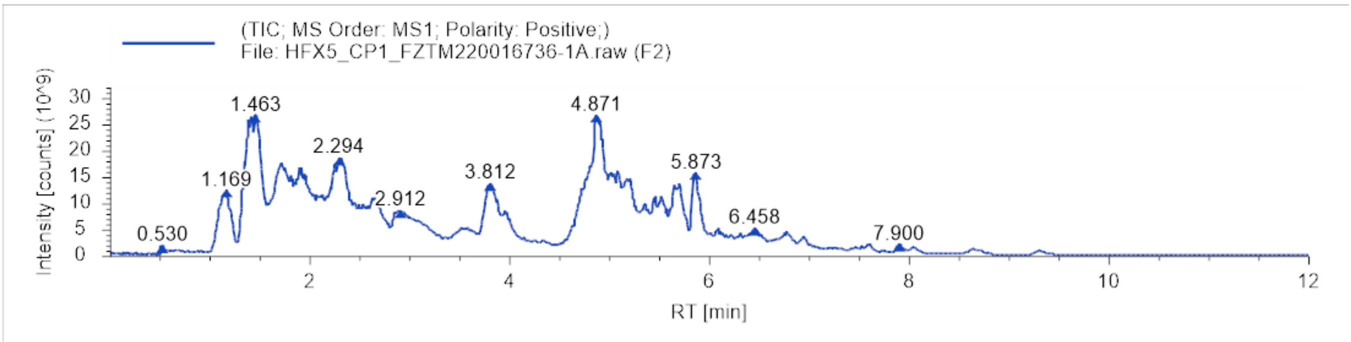

A

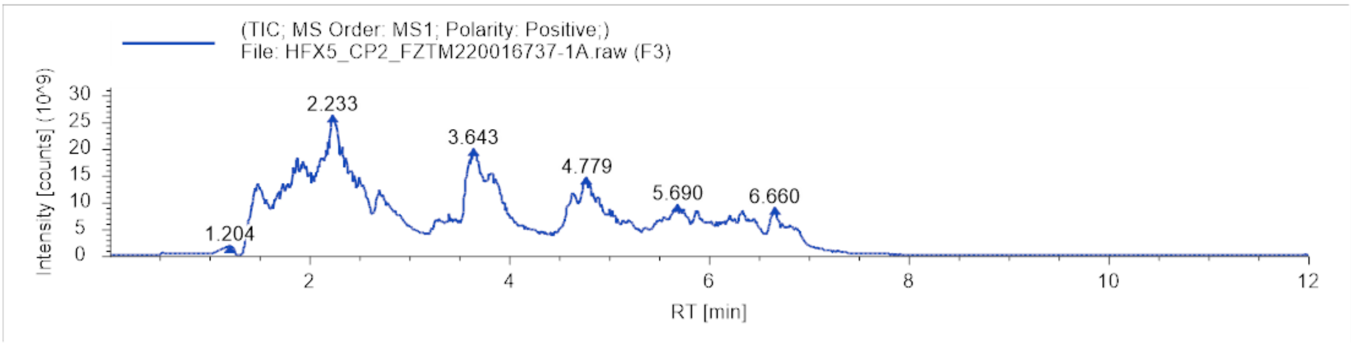

B

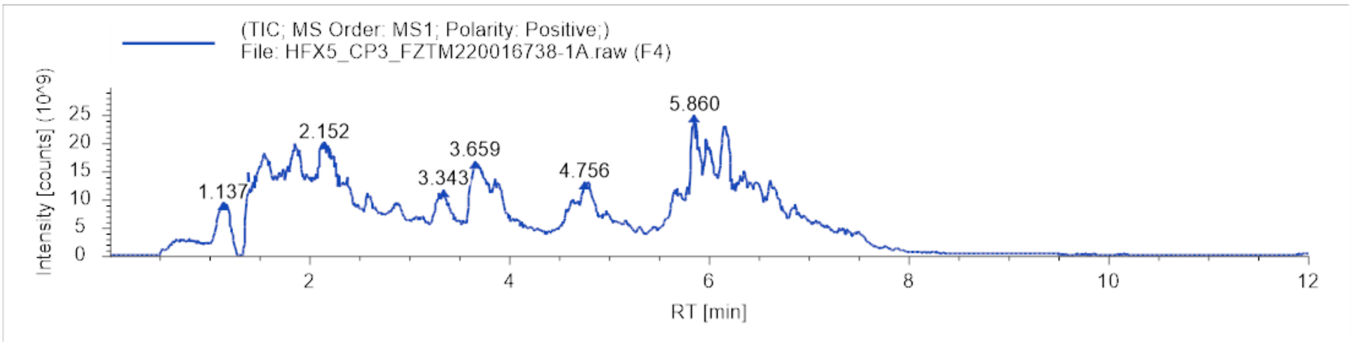

C

Figure S3. (**A**) Total ion chromatogram of Ethyl Acetate extract of Endophytic Fungi *Penicillium sclerotigenum* DCL06; (**B**) Total ion chromatogram of Ethyl Acetate extract of Endophytic Fungi *Diaporthe phaseolorum* DCL09; (**C**) Total ion chromatogram of Ethyl Acetate extract of Endophytic Fungi *Pestalotiopsis* sp. DCL44.

Table S1. The identification results of cultivable Endophytic Fungi from *Ageratina adenophora*.

Strain NO.	Tissue	Accession NO.	Number of bp Analyzed	Closest BLAST Match (GenBank Accession Number)	Identity (%)	Identification
DCL01	Leaf	MZ047481	550	*Aporospora terricola*（EU272515.1）	99	*Aporospora terricola*
DCL02	Leaf	MZ047482	564	*Alternaria* sp.（KC147581.1）	99	*Alternaria* sp.
DCL03	Leaf	MZ047483	564	*Alternaria alternata*（MT556702.1）	99	*Alternaria alternata*
DCL04	Leaf	MZ047484	590	*Aspergillus flavus*（MW522551.1）	99	*Aspergillus flavus*
DCL05	Leaf	MZ047485	630	*Trichoderma longibrachiatum*（MK910065.1）	99	*Trichoderma longibrachiatum*
DCL06	Leaf	MZ047486	590	*Penicillium sclerotigenum*（MK334370.1）	99	*Penicillium sclerotigenum*
DCL07	Leaf	MZ047487	542	*Fusarium oxysporum*（MT529348.1）	100	*Fusarium oxysporum*
DCL08	Leaf	MZ047488	543	*Didymella* sp.（KF911347.1）	99	*Didymella* sp.
DCL09	Leaf	MZ047489	572	*Diaporthe phaseolorum*（MW624472.1）	99	*Diaporthe phaseolorum*
DCL10	Leaf	MZ047490	576	*Aspergillus* sp.（MN905834.1）	100	*Aspergillus* sp.
DCL11	Leaf	MZ047491	565	*Alternaria alternata*（GQ169728.1）	100	*Alternaria alternata*
DCL12	Leaf	MZ047492	569	*Alternaria* sp.（MW332088.1）	99	*Alternaria* sp.
DCL13	Leaf	MZ047493	563	*Alternaria alternata*（MT446176.1）	99	*Alternaria alternata*
DCL14	Leaf	MZ047494	550	*Xylariaceae* sp.（MT446156.1）	100	*Xylariaceae* sp.
DCL15	Leaf	MZ047495	563	*Alternaria alternata*（MH368103.1）	99	*Alternaria alternata*
DCL16	Leaf	MZ047496	562	*Xylariaceae* sp.（MT446156.1）	99	*Xylariaceae* sp.
DCL17	Leaf	MZ047497	564	*Alternaria alternata*（KJ739877.1）	98	*Alternaria alternata*
DCL18	Leaf	MZ047498	565	*Xylariaceae* sp.（MT446156.1）	99	*Xylariaceae* sp.
DCL19	Leaf	MZ047499	528	*Fusarium graminearum*（MT228970.1）	99	*Fusarium graminearum*
DCL20	Leaf	MZ047500	580	*Penicillium* sp.（MW617884.1）	99	*Penicillium* sp.
DCL21	Leaf	MZ047501	562	*Xylariaceae* sp.（KC178658.1）	99	*Xylariaceae* sp.
DCL22	Leaf	MZ047502	537	*Cercospora* sp.（KC776152.1）	99	*Cercospora* sp.
DCL23	Leaf	MZ047503	535	*Cladosporium* sp.（KC178629.1）	100	*Cladosporium* sp.
DCL24	Leaf	MZ047504	566	*Alternaria* sp. (FJ196613.1)	99	*Alternaria* sp.
DCL25	Leaf	MZ047505	564	*Xylariaceae* sp.（MT446156.1）	99	*Xylariaceae* sp.
DCL26	Leaf	MZ047506	566	*Alternaria alternata* (MH368103.1)	99	*Alternaria alternata*
DCL27	Leaf	MZ047507	569	*Diaporthe novem* (MH299960.1)	99	*Diaporthe novem*
DCL28	Leaf	MZ047508	522	*Ampelomyces* sp. (KJ958371.1)	99	*Ampelomyces* sp.
DCL29	Leaf	MZ047509	560	*Diaporthe sojae (MK942675.1)*	99	*Diaporthe sojae*

DCL30	Leaf	MZ047510	569	*Colletotrichum* sp. (KC507287.1)	99	*Colletotrichum* sp.
DCL31	Leaf	MZ047511	619	*Trametes versicolo*r (MF475935.1)	99	*Trametes versicolor*
DCL32	Leaf	MZ047512	560	*Xylariaceae* sp. (MT446156.1)	99	*Xylariaceae* sp.
DCL33	Leaf	MZ047513	624	*Trametes hirsuta* (MF377430.1)	99	*Trametes hirsuta*
DCL34	Leaf	MZ047514	563	*Xylariaceae* sp. (KC178658.1)	99	*Xylariaceae* sp.
DCL35	Leaf	MZ047515	567	*Colletotrichum* sp.（MN121391.1）	99	*Colletotrichum* sp.
DCL36	Leaf	MZ047516	568	*Colletotrichum gloeosporioides*（KM044004.1）	100	*Colletotrichum gloeosporioides*
DCL37	Leaf	MZ047517	562	*Xylariaceae* sp .(MT446156.1)	99	*Xylariaceae* sp.
DCL38	Leaf	MZ047518	574	*Phomopsis* sp.（JQ954648.1）	99	*Phomopsis* sp.
DCL39	Leaf	MZ047519	561	*Alternaria alternata*（KJ739875.1）	99	*Alternaria alternata*
DCL40	Leaf	MZ047520	556	*Diaporthe novem*（MH299960.1）	99	*Diaporthe novem*
DCL41	Leaf	MZ047521	563	*Brunneomyces brunnescens*（MN592899.1）	99	*Brunneomyces brunnescens*
DCL42	Leaf	MZ047522	525	*Didymella sinensis*（MH257405.1）	99	*Didymella sinensis*
DCL43	Leaf	MZ047523	556	*Diaporthe novem*（MH299958.1）	99	*Diaporthe novem*
DCL44	Leaf	MZ066737	603	*Pestalotiopsis* sp.(KP120986.1)	99	*Pestalotiopsis* sp.
DCL45	Leaf	MZ066738	536	*Stagonosporopsis cucurbitacearum*（JQ936150.1）	99	*Stagonosporopsis cucurbitacearum*
DCL46	Leaf	MZ066739	565	*Alternaria alternata*（MT556702.1）	99	*Alternaria alternata*
DCL47	Leaf	MZ066740	601	*Pestalotiopsis kenyana*（MK910059.1）	99	*Pestalotiopsis kenyana*
DCL48	Leaf	MZ066741	599	*Pestalotiopsis* sp.（KF412648.1）	99	*Pestalotiopsis* sp.
DCL49	Leaf	MZ066742	566	*Colletotrichum gloeosporioides*（KF577905.1）	99	*Colletotrichum gloeosporioides*
DCL50	Leaf	MZ066743	531	*Botrytis cinerea*（MH992149.1）	99	*Botrytis cinerea*
DCL51	Leaf	MZ066744	532	*Botrytis fabae*（MN589851.1）	99	*Botrytis fabae*
DCL52	Leaf	MZ066745	567	*Colletotrichum siamense*（MN296056.1）	99	*Colletotrichum siamense*
DCL53	Leaf	MZ066746	537	*Fusarium oxysporum*（MT529616.1）	99	*Fusarium oxysporum*
DCL54	Leaf	MZ066747	536	*Botrytis cinerea* (KR055050.1)	99	*Botrytis cinerea*
DCL55	Leaf	MZ066748	578	*Colletotrichum godetiae*（MT133295.1）	99	*Colletotrichum godetiae*
DCL56	Leaf	MZ066749	571	*Diaporthe ambigua*(MF319487.1)	99	*Diaporthe ambigua*
DCS01	Stem	MZ047524	565	*Alternaria alternata*(KJ739877.1)	99	*AltFernaria alternata*
DCS02	Stem	MZ047525	546	*Stagonosporopsis cucurbitacearum* (JQ936326.1)	99	*Stagonosporopsis cucurbitacearum*
DCS03	Stem	MZ047526	571	*Alternaria dauci* (JQ936188.1)	99	*Alternaria dauci*
DCS04	Stem	MZ047527	566	*Alternaria alternata*(GQ169728.1)	100	*Alternaria alternata*

DCS05	Stem	MZ047528	550	*Alternaria* sp. (KC139505.1)	100	*Alternaria* sp.
DCS06	Stem	MZ047529	560	*Alternaria alternata*（MT446076.1）	99	*Alternaria alternata*
DCS07	Stem	MZ047530	534	*Phoma* sp.（MK299419.1）	99	*Phoma* sp.
DCS08	Stem	MZ047531	605	*Trichoderma tomentosum*（AY605737.1）	99	*Trichoderma tomentosum*
DCS09	Stem	MZ047532	610	*Trichoderma tomentosum*（EU280083.1）	99	*Trichoderma tomentosum*
DCS10	Stem	MZ047533	543	*Fusarium verticillioides* (KX196811.1)	99	*Fusarium verticillioides*
DCS11	Stem	MZ047534	562	*Alternaria alternata*(KJ739876.1)	99	*Alternaria alternata*
DCS12	Stem	MZ047535	563	*Penicillium* sp. (MW617884.1)	100	*Penicillium* sp.
DCS13	Stem	MZ047536	566	*Xylariaceae* sp. (MT446156.1)	100	*Xylariaceae* sp.
DCS14	Stem	MZ047537	534	*Cladosporium* sp. (KC339771.1)	99	*Cladosporium* sp.
DCS15	Stem	MZ047538	560	*Xylariaceae* sp. (MT446156.1)	99	*Xylariaceae* sp.
DCS16	Stem	MZ047539	545	*Alternaria alternata*（KJ605840.1）	100	*Alternaria alternata*
DCS17	Stem	MZ047540	547	*Alternaria alternata*（MT446184.1）	100	*Alternaria alternata*
DCS18	Stem	MZ047541	562	*Alternaria alternata* (MH368103.1)	99	*Alternaria alternata*
DCS19	Stem	MZ047542	576	*Penicillium* sp. (KF367551.1)	99	*Penicillium* sp.
DCS20	Stem	MZ047543	570	*Colletotrichum liriopes*(MN589679.1)	99	*Colletotrichum liriopes*
DCS21	Stem	MZ047544	550	*Cladosporium* sp. (KC339771.1)	99	*Cladosporium* sp.
DCS22	Stem	MZ047545	537	*Fusarium oxysporum*(MN018399.1)	100	*Fusarium oxysporum*
DCS23	Stem	MZ047546	532	*Cladosporium* sp. (KC339771.1)	99	*Cladosporium* sp.
DCS24	Stem	MZ047547	548	*Cladosporium* sp. (KC178629.1)	99	*Cladosporium* sp.
DCS25	Stem	MZ047548	600	*Trichothecium roseum*(MW440515.1)	99	*Trichothecium roseum*
DCS26	Stem	MZ047549	577	*Penicillium ochrochloron* (MK720828.1)	99	*Penicillium ochrochloron*
DCS27	Stem	MZ047550	578	*Trichoderma sulphureum*(MT529378.1)	100	*Trichoderma sulphureum*
DCS28	Stem	MZ047551	544	*Alternaria alternata*(MT556702.1)	100	*Alternaria alternata*
DCS29	Stem	MZ047552	560	*Diaporthe sojae*(MK942675.1)	99	*Diaporthe sojae*
DCS30	Stem	MZ047553	598	*Trichoderma tomentosum*(MN148667.1)	99	*Trichoderma tomentosum*
DCS31	Stem	MZ047554	576	*Trichoderma sulphureum*(MT529378.1)	100	*Trichoderma sulphureum*
DCS32	Stem	MZ047555	545	*Alternaria alternata* (MT446184.1)	100	*Alternaria alternata*
DCR01	Root	MZ047556	574	*Penicillium commune*(KY552638.1)	99	*Penicillium commune*
DCR02	Root	MZ047557	541	*Didymella* sp. (KF911347.1)	99	*Didymella* sp.
DCR03	Root	MZ047558	582	*Penicillium concentricum* (EU551180.1)	99	*Penicillium concentricum*

DCR04	Root	MZ047559	566	*Alternaria alternata*(GQ169728.1)	100	*Alternaria alternata*
DCR05	Root	MZ047560	578	*Phomopsis* sp. (HQ832815.1)	99	*Phomopsis* sp.
DCR06	Root	MZ047561	539	*Fusarium oxysporum*(GU724514.1)	99	*Fusarium oxysporum*
DCR07	Root	MZ047562	544	*Fusarium kyushuense*(MK247795.1)	100	*Fusarium kyushuense*
DCR08	Root	MZ047563	561	*Diaporthe novem*(MH299960.1)	99	*Diaporthe novem*
DCR09	Root	MZ047564	564	*Fusarium solani*（KX034335.1）	99	*Fusarium solani*
DCR10	Root	MZ047565	625	*Mucor fragilis* *（*MN069560.1*）*	99f	*Mucor fragilis*
DCR11	Root	MZ047566	539	*Didymella* sp.（HM068373.1）	99	*Didymella* sp.
DCR12	Root	MZ047567	560	*Fusarium solani*（MK734064.1）	99	*Fusarium solani*
DCR13	Root	MZ047568	551	*Plectosphaerella* sp.（MN275877.1）	99	*Plectosphaerella* sp.
DCR14	Root	MZ047569	629	*Mucor fragilis*（MN069560.1）	99	*Mucor fragilis*
DCR15	Root	MZ047570	563	*Alternaria alternata*（MH368103.1）	99	*Alternaria alternata*
DCR16	Root	MZ047571	539	*Fusarium solani*（MT560338.1）	99	*Fusarium solani*
DCR17	Root	MZ047572	552	*Gregarithecium curvisporum* (NR154049.1)	92	*Gregarithecium curvisporum*
DCR18	Root	MZ047573	579	*Byssochlamys spectabilis*（MN547409.1）	99	*Byssochlamys spectabilis*
DCR19	Root	MZ047574	544	*Cladosporium oxysporum*（MG840724.1）	100	*Cladosporium oxysporum*
DCR20	Root	MZ047575	554	*Fusarium acuminatum*（MK583544.1）	99	*Fusarium acuminatum*
DCR21	Root	MZ047576	525	*Cladosporium* sp.（FJ176478.1）	100	*Cladosporium* sp.
DCR22	Root	MZ047577	599	*Trichoderma gamsii*（GQ351597.1）	99	*Trichoderma gamsii*
DCR23	Root	MZ047578	561	*Alternaria* sp.（KC110624.1）	100	*Alternaria* sp.
DCR24	Root	MZ047579	548	*Cladosporium* sp.（KC178629.1）	99	*Cladosporium* sp.
DCR25	Root	MZ047580	564	*Xylariaceae* sp.（MT446156.1）	99	*Xylariaceae* sp.
DCR26	Root	MZ047581	579	*Penicillium* sp. *(KF367512.1)*	99	*Penicillium* sp.
DCR27	Root	MZ047582	560	*Diaporthe ambigua* (MH864620.1)	99	*Diaporthe ambigua*
DCR28	Root	MZ047583	612	*Trichoderma* sp. (KY086243.1)	99	*Trichoderma* sp.
DCR29	Root	MZ066750	538	*Fusarium oxysporum* (MT529890.1*)*	99	*Fusarium oxysporum*
DCR30	Root	MZ066751	536	*Fusarium oxysporum* (MT529890.1*)*	99	*Fusarium oxysporum*
DCR31	Root	MZ066752	538	*Fusarium oxysporum* (GU724514.1 *)*	99	*Fusarium oxysporum*
DCR32	Root	MZ066753	536	*Stagonosporopsis cucurbitacearum*（JQ936326.1）	99	*Stagonosporopsis cucurbitacearum*
DCR33	Root	MZ066754	549	*Nigrospora sphaerica*（MT466514.1）	99	*Nigrospora sphaerica*
DCR34	Root	MZ066755	586	*Pestalotiopsis oryzae*（MT123044.1）	99	*Pestalotiopsis oryzae*

DCR35	Root	MZ066756	571	*Diaporthe novem*（MH299960.1）	99	*Diaporthe novem*
DCR36	Root	MZ066757	572	*Diaporthe ambigua*（MF319487.1）	99	*Diaporthe ambigua*
